# Epidemiology, Patterns of treatment, and Mortality of Pediatric Trauma Patients in Japan

**DOI:** 10.1038/s41598-018-37579-3

**Published:** 2019-01-29

**Authors:** Makoto Aoki, Toshikazu Abe, Daizoh Saitoh, Kiyohiro Oshima

**Affiliations:** 1 Department of Emergency Medicine, Gunma University Graduate School of Medicine, Gunma, Japan; 2 Department of General Medicine, Juntendo University, Tokyo, Japan; 3 Department of Health Services Research, Faculty of Medicine, University of Tsukuba, Ibaraki, Japan; 4 Department of Traumatology and Emergency Medicine, National Defense Medical College, Saitama, Japan

## Abstract

Limited information exists regarding the epidemiology, patterns of treatment, and mortality of pediatric trauma patients in Japan. To evaluate the characteristics and mortality of pediatric trauma patients in Japan, especially in traffic accidents. This was a retrospective cohort study between 2004 and 2015 from a nationwide trauma registry in Japan. Pediatric trauma patients divided into four age groups: <1 years; 1 ≤ 5 years; 6 ≤ 10 years; and 11 ≤ 15 years. Data on patients’ demographics, trauma mechanism and severity, treatments and in-hospital mortality were analyzed between the groups. There were 15,441 pediatric trauma patients during the study period. Among 15,441 pediatric patients, 779 belonged to the <1 year age group, 3,933 to the 1 ≤ 5 years age group, 5,545 to the 6 ≤ 10 age group, and 5,184 to the 11 ≤ 15 years age group. Male injuries (69%) were more frequent than female injuries. Head injuries (44%) were the most frequent and severe. Traffic accidents were the leading cause of trauma (44%). Overall in-hospital mortality was 3.9% and emergency department mortality was 1.4%. In-hospital mortality was 5.3%, 4.7%, 3.0% and 4.0% for the <1 year, 1 ≤ 5 years, 6 ≤ 10 years, and 11 ≤ 15 years age groups respectively. A total of 57% of all trauma deaths were before or upon arrival at hospital. Traffic accidents for the <1 year age group was the highest category of mortality (15%). The overall in-hospital mortality of Japanese pediatric trauma patients was 3.9% based on the nationwide trauma registry of Japan. The main cause of severe trauma was traffic accidents, especially in patients <1 year of age whose mortality was 15%.

## Introduction

Trauma has been a major cause of death in young populations^1^. The burden of trauma ranges from physiological^2^ to economic^3^ causes, which could be a serious negative effect especially in developed countries that already have decrease in the number of children. Therefore, it is important to know the epidemiology, the patterns of treatment, and mortality of pediatric trauma.

In Japan, the Japan Advanced Trauma Evaluation and Care was introduced in 2002 and since then mortality has decreased^4,5^. However, the last report showed that the mortality of pediatric trauma patients did not improve^4^ and there remained limited information about pediatric trauma.

The current mortality, the main causes of mortality among pediatric trauma patients in Japan, especially for traffic accidents, the mechanisms of traffic accidents associated with mortality, and the major affected organs are currently not known. The understanding of these issues could lead to trauma prevention. In addition, the evaluation of the patterns of treatment and the main causes of mortality could lead to improvements in trauma care. Therefore, we undertook a large observational study to gain insight into the epidemiology, the patterns of treatment, and the trends of in-hospital mortality of pediatric trauma in Japan.

## Methods

### Study design

This study was a multicenter, retrospective cohort study using the Japan Trauma Data Bank (JTDB) data between 2004 and 2015. JTDB is a nationwide trauma registry established in 2003 by the Japanese Association for the Surgery of Trauma and the Japanese Association for Acute Medicine to improve and ensure the quality of trauma care in Japan^6^. During the study period, 260 hospitals including 95% of tertiary emergency medical centers in Japan participated in the JTDB. The JTDB collects 92 data elements related to patient and hospital information such as patient demographics, premorbid medical conditions, vital signs, abbreviated injury scale (AIS) score, injury severity score (ISS), in-hospital procedures, as well as in-hospital and in-emergency department (ED) mortality^6^. The primary outcome of this investigation was in-hospital mortality and the secondary outcome was in-ED mortality.

### Patient Selection and Patient Categories

All pediatric trauma patients aged <16 years were enrolled. Exclusion criteria were patients whose age was missing. As expected, the mortality, mechanism of injury, cause of injury, and the normal vital signs differed by age, therefore, we divided the patients into four age groups including: aged <1 year, aged 1 ≤ 5 years, aged 6 ≤ 10 years, and aged 11 ≤ 15 years based on previous articles^7–10^.

### Statistical analysis

Continuous variables were expressed as medians and interquartile range (IQR). Categorical variables were expressed as counts and percentages. Subgroup analyses regarding traffic accidents was performed since it has been the main cause of the mortality among pediatric patients in Japan^11^. Traffic accidents were classified into motor vehicle collision, bike, bicycle, and pedestrian. We also analyzed associations between traffic accident mechanisms and mortality in each age group. In addition, we performed subgroup analysis of deceased children. To evaluate whether the pediatric trauma mortality could be reduced or not between the early and late periods, patients were divided into two groups: 2004–2010 and 2011–2015. The comparisons of each categorical variable between groups were performed using the chi-squared test and the comparisons of each continuous variable between groups were performed using the Mann-Whitney U test.

Statistical significance was defined as a two-sided p value < 0.05 in all statistical analyses. All statistical analyses were performed using the IBM SPSS Statistics version 25.0 (SPSS Inc, Chicago, Illinois, USA).

## Results

### Patient mortality

We analyzed 16,038 pediatric trauma patients aged <16 years from a total of 236,698 trauma patients. A total of 15,441 pediatric trauma patients remained after exclusion of patients with age data missing (n = 597). There were 779 patients aged <1 years, 3,933 aged 1 ≤ 5 years, 5545 ≤ 10 years, and 5,184 aged 11 ≤ 15 years. Males accounted for 69% of the study group. The 11 ≤ 15 age group had the highest percentage of boys (74%) (Table 1). Overall in-hospital mortality was 3.9% and ED mortality was 1.4%. In-hospital mortality was 5.3%, 4.7%, 3.0%, and 4.0% for the <1 year, 1 ≤ 5 years, 6 ≤ 10 years, and 11 ≤ 15 years age groups (Table 1). A total of 86% of the patients were discharged to go home and 13% were derived to another hospital. The median number of days of hospitalization was 5.

**Table 1:** Characteristics of pediatric trauma patients divided into age groups (years).

Variable	Age	Age	Age	Age
	(<1)	(1–5)	(6–10)	(11–15)
	n = 779	n = 3933	n = 5545	n = 5184
Gender (male)	492/779 (63%)	2481/3931 (63%)	3884/5544 (70%)	3847/5182 (74%)
Cause of injury				
Traffic accident	77/727 (11%)	1127/3784 (30%)	2917/5357 (54%)	2419/4998 (48%)
Fall	379/727 (52%)	1765/3784 (47%)	1720/5357 (32%)	1236/4998 (25%)
Sport	0/727 (0%)	21/3784 (0.6%)	269/5357 (5.0%)	893/4998 (18%)
Other blunt trauma	89/727 (12%)	363/3784 (9.6%)	304/5357 (5.7%)	300/4998 (6.0%)
Penetrate	10/727 (1.4%)	101/3784 (2.7%)	65/5357 (1.2%)	82/4998 (1.6%)
Burn	172/727 (24%)	407/3784 (11%)	82/5357 (1.5%)	68/4998 (1.4%)
AIS				
Head (n = 6,836)	4 (3–4)	3 (2–4)	3 (2–4)	3 (2–4)
Face (n = 2,696)	1 (1–1)	1 (1–1)	1 (1–2)	1 (1–2)
Neck (n = 109)	2 (2–2)	2 (1–3)	1 (1–2)	1 (1–2)
Thorax (n = 2,082)	3 (3–4)	3 (3–4)	3 (3–4)	3 (3–4)
Abdomen and pelvis (n = 1,866)	2 (1–3)	2 (2–3)	2 (2–3)	3 (2–3)
Cervical spine (n = 630)	3 (2–3)	3 (2–5)	2 (1–3)	2 (2–3)
Upper extremity (n = 3,841)	2 (1–2)	2 (2–3)	2 (2–3)	2 (2–3)
Lower extremity (n = 3,493)	3 (3–3)	3 (1–3)	2 (1–3)	2 (1–3)
Others (n = 1,401)	3 (1–3)	2 (1–3)	1 (1–1)	1 (1–1)
ISS	16 (9–16)	9 (4–16)	9 (5–16)	9 (5–17)
FAST				
Positive	7/676 (1.0%)	122/3577 (3.4%)	297/5125 (5.8%)	313/4778 (6.6%)
Negative	189/676 (28%)	1547/3577 (43%)	3053/5125 (60%)	2799/4778 (59%)
Not conducted	480/676 (71%)	1908/3577 (53%)	1775/5125 (35%)	1666/4778 (35%)
Pan-scan CT	50 (6.4%)	639 (16%)	1251 (23%)	1299 (25%)
Treatment				
Craniotomy	53 (6.8%)	118 (3.0%)	191 (3.4%)	190 (3.7%)
Thoracotomy	0 (0%)	21 (0.5%)	23 (0.4%)	30 (0.6%)
Celiotomy	0 (0%)	29 (0.7%)	74 (1.3%)	88 (1.7%)
TAE	0 (0%)	22 (0.6%)	81 (1.5%)	133 (2.6%)
Blood transfusion	64/725 (8.8%)	197/3677 (5.4%)	278/5254 (5.3%)	310/4916 (6.3%)
Mortality in hospital	34/637 (5.3%)	142/2994 (4.7%)	141/4742 (3.0%)	179/4501 (4.0%)
Mortality in ED	11/775 (1.4%)	68/3922 (1.7%)	56/5533 (1.0%)	81/5167 (1.6%)
Discharged place				
Home	493/585 (84%)	2490/2826 (88%)	4016/4574 (88%)	3571/4291 (83%)
Another hospital	70/585 (12%)	303/2826 (11%)	531/4574 (12%)	698/4291 (16%)
Other	22//585 (3.8%)	33/2826 (1.2%)	27/4574 (0.6%)	22/4291 (0.5%)
Length of hospital stay	5 (1–13)	3 (1–11)	5 (1–13)	7 (2–17)

IQR, interquartile range; sBP, systolic blood pressure; HR, heart rate; RR, respiratory rate; GCS. Glasgow coma scale; RTS, revised trauma score; AIS, abbreviated injury scale; ISS, injury severity score; TRISS, trauma and injury severity score; FAST, focused assessment with sonography for trauma; TAE, transcatheter arterial embolization; ED, emergency department.

Missing: Gender = 5, Cause of Injury = 575, ISS = 1416, FAST = 1285, Blood transfusion = 869, Mortality in hospital = 2567, Mortality in ED = 44, Discharged place = 2669 and Length of hospital stay = 2637.

### Causes of injury and injured organs

Trauma caused by falls was the most frequent, accounting for 52% and 47% of all traumas in the <1 year and the 1 ≤ 5 years age groups respectively (Table 1). Traffic accidents were the most frequent cause of trauma among the 6 ≤ 10 years and the 11 ≤ 15 years age groups accounting for 54% and 48%, respectively (Tables 1 and 2). Burns were frequent in the <1 year age group (24%) and sport was frequent in the aged 11 ≤ 15 age group (18%).

**Table 2:** Characteristics of pediatric patients with traffic accidents divided into age group (years).

Variable	Age	Age	Age	Age
	(<1)	(1–5)	(6–10)	(11–15)
	n = 77	n = 1127	n = 2917	n = 2419
Gender (male)	46/77 (60%)	729/1127 (65%)	2018/2916 (69%)	1705/2419 (71%)
Cause of injury				
Traffic accident				
Motor vehicle	69/77 (90%)	288/1127 (26%)	278/2917 (9.5%)	191/2419 (7.9%)
Bike	0/77 (0%)	7/1127 (0.6%)	30/2917 (1.0%)	246/2419 (10%)
Bicycle	4/77 (5.2%)	166/1127 (15%)	1151/2917 (40%)	1579/2419 (65%)
Pedestrian	4/77 (5.2%)	666/1127 (59%)	1458/2917 (50%)	403/2419 (17%)
AIS (median [IQR])				
Head (n = 3704)	4 (3–4)	3 (2–4)	3 (2–4)	3 (2–4)
Face (n = 1661)	1 (1–1)	1 (1–1)	1 (1–1)	1 (1–2)
Neck (n = 52)	N.A	1 (1–1)	1 (1–2)	1 (1–2)
Thorax (n = 1401)	3 (3–4)	3 (3–4)	3 (3–4)	3 (3–4)
Abdomen and pelvis (n = 1083)	2 (1–3)	2 (2–3)	2 (2–3)	2 (2–3)
Cervical spine (n = 264)	3 (2–3)	3 (2–6)	2 (2–3)	2 (1–3)
Upper extremity (n = 1486)	N.A	2 (1–2)	2 (1–2)	2 (1–2)
Lower extremity (n = 2278)	3 (2–3)	2 (1–3)	2 (1–3)	2 (1–3)
Others (n = 545)	N.A	1 (1–1)	1 (1–1)	1 (1–1)
ISS (median [IQR])	16 (9–17)	10 (5–20)	10 (8–18)	10 (8–20)
FAST				
Positive	4/74 (5.4%)	74/1061 (7.0%)	167/2768 (6.0%)	143/2301 (6.2%)
Negative	43/74 (58%)	729/1061 (69%)	2195/2768 (79%)	1817/2301 (79%)
Not conducted	27/74 (37%)	258/1061 (24%)	406/2768 (15%)	341//2301 (15%)
Pan-scan CT	14 (18%)	341 (30%)	975 (33%)	836 (35%)
Treatment				
Craniotomy	5 (6.5%)	25 (2.2%)	120 (4.1%)	124 (5.1%)
Thoracotomy	0 (0%)	13 (1.2%)	16 (0.5%)	14 (0.6%)
Celiotomy	0 (0%)	21 (1.9%)	50 (1.7%)	35 (1.4%)
TAE	0 (0%)	14 (1.2%)	51 (1.7%)	59 (2.4%)
Blood transfusion	12/73 (16%)	102/1095 (9.3%)	205/2815 (7.3%)	166/2337 (7.1%)
Mortality in ED	3/77 (3.9%)	48/1127 (4.3%)	44/2914 (1.5%)	27/2416 (1.1%)
Mortality in hospital	9/60 (15%)	85/946 (9.0%)	106/2537 (4.2%)	80/2144 (3.7%)
Discharged place				
Home	41/51 (80%)	719/850 (85%)	2034/2414 (84%)	1674/2048 (82%)
Another hospital	9/51 (18%)	124/850 (15%)	367/2414 (15%)	367/2048 (18%)
Other	1/51 (2.0%)	7/850 (0.8%)	13/2414 (0.5%)	7/2048 (0.3%)
Length of hospital stay	5 (1–13)	5 (1–14)	8 (2–18)	9 (2–19)

IQR, interquartile range; sBP, systolic blood pressure; HR, heart rate; RR, respiratory rate; GCS. Glasgow coma scale; RTS, revised trauma score; AIS, abbreviated injury scale; ISS, injury severity score; TRISS, trauma and injury severity score; FAST, focused assessment with sonography for trauma; TAE, transcatheter arterial embolization; ED, emergency department.

Missing: Gender = 1, ISS = 448, FAST = 336, Blood transfusion = 220, Mortality in ED = 6, Mortality in hospital = 853, Discharged place = 897 and Length of hospital stay = 859.

The head was the most frequent injured body part with 44% of the pediatric trauma patients suffering from head trauma (Table 1). A total of 58% of the <1 year age group had head trauma. The <1 year age group had the highest abbreviated injury scale (AIS) of the head region and had the highest injury severity score (ISS) compared to the other age groups. Regarding chest trauma, the percentages increased with age: 3.0% for the <1 year age group, 12% for the 1 ≤ 5 years age group, 14% for the 6 ≤ 10 years age group, and 16% for the 11 ≤ 15 years age group.

### Diagnosis and treatment procedures

The older the patients the higher the use of sonography of trauma (FAST) and pan-scan CT for assessment (Table 1). Craniotomy was the sole treatment procedure for the <1 year age group (6.8%). The percentages of thoracotomy, celiotomy, and transcatheter arterial embolization (TAE) increased with age. Regarding blood transfusions, 8.8% of the <1 year patients had blood transfusions.

### Traffic accidents sub analysis

More than half of the pediatric patients suffering traffic accidents were males and the percentages of boys increased with age (Table 2). The most frequent traffic accident in the <1 year age group was motor vehicle, walking in the 1 ≤ 5 and the 6 ≤ 10 age groups, while riding a bicycle in the 11 ≤ 15 age group.

### Deceased children sub analysis

Table 3 shows the mortalities of each mechanism of trauma. The <1 year age group had the highest mortality caused by traffic accidents (15%) and the 11 ≤ 15 year age group had the highest mortality caused by falls (5.8%) and burns (12%).

**Table 3:** Mortalities of pediatric trauma patients with cause of injury divided into age group (years).

Variable	Age	Age	Age	Age
	(<1)	(1–5)	(6–10)	(11–15)
All cause (n = 12874)	34/637 (5.3%)	142/2994 (4.7%)	141/4742 (3.0%)	179/4501 (4.0%)
Traffic accident (n = 5687)	9/60 (15%)	85/946 (9.0%)	106/2537 (4.2%)	80/2144 (3.7%)
Fall (n = 4279)	9/327 (2.8%)	30/1341 (2.2%)	23/1525 (1.5%)	63/1086 (5.8%)
Burn (n = 610)	2/138 (1.4%)	7/342 (2.0%)	1/70 (1.4%)	7/60 (12%)

Missing: All cause = 2567, Traffic accident = 853, Fall = 821 and Burn = 119.

Among all age groups with in-hospital deaths, the highest AIS region was the head (Supplemental Table 1). Among in-hospital deaths, 57% were prehospital and/or arrival cardiac pulmonary arrests (CPA) (Supplemental Table 2). The highest AIS region was also the head in the prehospital and/or arrival CPA patients; however, there were no craniotomies. Thoracotomy and/or celiotomy were done in patients over 1 year of age.

### Differences between early period 2004–2010 and late period 2011–2015

Between these periods, percentage of male pediatric trauma patients was significantly higher in Early period (p = 0.025) (Supplemental Table 3). As to cause of injury, prevalence of traffic accident was significantly decreased (Early period 48% vs Late period 42%; p < 0.001) and severe head, chest and abdominal injury were decreased (severe head injury: Early period 23% vs Late period 16%; p < 0.001, severe chest injury: Early period 5.0% vs Late period 4.0%; p = 0.004 and severe abdominal injury: Early period 2.0% vs Late period 1.5%; p = 0.038, respectively). As to the treatment, craniotomy, thoracotomy, celiotomy and blood transfusion were decreased. In addition, both the outcomes of mortality in ED and mortality in hospital were significantly lower in Late period (Mortality in ED: Early period 1.8% vs Late period 1.2%; p = 0.002 and Mortality in hospital: Early period 5.5% vs Late period 3.0%; p < 0.001)

## Discussion

### Brief summary

This study investigated for the first time the epidemiology of pediatric trauma patients using a nationwide cohort registry in Japan. We found that overall, in-hospital mortality was 3.9% and children aged <1 year had the highest percentage of in-hospital mortality, especially caused by traffic accidents with an in-hospital mortality of 15%.

### Characteristics of the pediatric trauma patients

Boys tended to suffer from trauma more than girls across all age groups. The percentages of boys with trauma increased as the age group increased, in agreement with previous studies^12,13^. This is because boys are generally likely to be exposed to risky activities and/or be more careless^14^. Regarding the causes of trauma, 52% of children aged <1 year suffered from falls and accounted for 2.8% mortality which is more frequent than previously reported^15^. However, a lower fall rate accounted for severe TBI. As to another finding of the causes of trauma, 24% of children aged <1 year suffered from burns. Pediatric burns can only occur due to careless behavior; however, this high prevalence could be attributed to abuse. A previous study reported that about 10% of burn cases were suspected abuse cases^16^. Concerning to abuse and/or neglect, one observational study from Japan reported that about half of the traumatic intracranial hemorrhage was caused by abuse^17^. Though abuse data was not registered in JTDB and the association between pediatric burns and abuse could not investigated, the high prevalence of pediatric burns in children <1 year clinicians suspected abuse and/or neglect. Children aged 11 ≤ 15 years had 15% of sport-related injuries. In- hospital mortality caused by sport-related injury was only 1.0%. A previous study reported that sports-related injuries were associated with spinal injury^18,19^ Here, we found that 6.5% of sport-related injury patients had cervical spine injury.

Regarding injury location, the most frequent and fatal injury was head trauma. Head trauma has been the leading cause of death and disability^20^ in spite of concomitant injuries in other areas such as the thorax that was reported to be associated with greater morbidity^21^.

Regarding diagnostic procedures, pan-scan CT is indicated based not only on the severity of the trauma but also on the age of the patient. Children are more radiosensitive and have a higher risk of carcinogenesis^22^, therefore clinicians are hesitant to perform pan-scan CT in young children. Treatment procedures frequencies such as operations and interventional radiology are low, which may be due to technical difficulties^23^, and thus conservative management is generally accepted in pediatric trauma patients^24,25^. On the other hand, the trend of diagnostic choice between early and late phase demonstrated that the use of pan-scan CT increased regardless of the reduction of severe pediatric injury (Supplemental Table 3). The usefulness of pan-scan CT for pediatric trauma is still controversial^26^; however, this reveals the future challenges of increasing survival rate. Regarding pediatric trauma in clinical practice, specialized centers for pediatric trauma were associated with reduced morality^27^. Therefore, to increase survival rate, we may need specialized pediatric trauma centers^27^.

Traffic accidents accounted for about half of the causes of trauma with a higher prevalence of males. In children aged <1 year, 90% of traffic accidents were motor vehicle accidents. Naturally, parent’s attentiveness in this case is important^28^. In addition, prevention programs were thought to have an important role in this age category^29^. The type of traffic accidents was different depending on the age groups. The leading type of accident was pedestrian in children aged 1 ≤ 5 and 6 ≤ 10 years. However, the prevalence of bicycle-related traffic accidents increased as the age group increased and was the leading cause in the 11 ≤ 15 years age group^30^.

### Mortality of pediatric trauma patients

Trauma has been a leading cause of death in both the developing and developed countries^1,31^. In-hospital mortality of pediatric trauma patients was reported to vary from 0.3% to 8.5% in the USA, Canada, Afghanistan, Iraq, and Europe^32–38^. In this report, the overall in-hospital mortality was 3.9% between 2004 and 2015 and mortality decreased to 2.5% for the 2011–2015 period similar or a little higher than that of other developed countries^32,33,35–38^. As age decreased, mortality has been previously reported to increase^39^, in agreement with this study. However, a mortality of 15% in children <1 year caused by traffic accident is frightening. On the other hand, mortality decreased to 7.7% in the last 5 years, although was nevertheless high compared to previous reports^7,36^. The leading cause of mortality was head trauma; however, in this study the incidence of emergency procedures and treatment such as craniotomy were limited. Severely injured children have been reported to have higher incidence of early mortality compared to adults. Given that 57% of casualties developed prehospital and/or arrival cardiac pulmonary arrest (CPA), there were less alive patients to undertake emergency procedures and/or treatment. This highlights the necessity for trauma prevention programs. The main reason of the mortality reduction between 2004–2010 and 2011–2015 was trauma prevention and social safety development^5,40^. The characteristics of the pediatric trauma patients between these periods demonstrated the reduction of the percentage of traffic accidents and severe pediatric trauma (Supplemental Table 3). Enforcing road traffic laws and proper use of child restraint seats could contribute to the reduction of pediatric deaths due to trauma^41^.

Within mortality caused by the other mechanisms, children aged 11 ≤ 15 years had higher mortality caused by falls and burns. Unfortunately, about 70% of deaths caused by falls and 29% of deaths caused by burns among the 11 ≤ 15 years age group were due to suicide. Falls from high places and burns are common methods of suicide^42^ and both causes of mortality could be increasing in the future^43^, indicating that anti suicide programs are needed.

This study has several limitations. First, this was a multicenter retrospective study using JTDB and 95% of tertiary emergency medical centers in Japan participated in the JTDB. Patients registration were committed to each facility and not all of the pediatric patients were necessarily registered in the JTDB. Second, there were some important variables missing. Lack of cause of injury and outcomes could impact the results.

## Conclusion

We analyzed the epidemiological characteristics of pediatric trauma patients in Japan. The overall in-hospital mortality was 3.9%. The leading cause of trauma was traffic accidents and children below 1 year of age had a 15% of mortality. Trauma is still the leading cause of death.

## Declarations

### Ethical Approval and Consent to participate

This study was approved by the medical ethics committee of the Gunma University Hospital. Because of the anonymous and retrospective nature of the study, the need for informed consent was waived.

## Supplementary information

Dataset 1
