# Supplementary material for: Epidemiology, Patterns of treatment, and Mortality of Pediatric Trauma Patients in Japan
Source: Sci Rep. 2019 Jan 29;9:917. doi: 10.1038/s41598-018-37579-3 (PMC6351578; doi:10.1038/s41598-018-37579-3)
Supplement: Supplementary file 1 — Dataset 1 [file 41598_2018_37579_MOESM1_ESM.docx]

Supplementary Data

**Title:** Epidemiology, Patterns of treatment, and Mortality of Pediatric Trauma Patients in Japan

**Authors:** Makoto Aoki, MD, PhD^1)^, Toshikazu Abe, MD, MPH^2,3)^,

Daizoh Saitoh, MD, PhD^4)^, Kiyohiro Oshima, MD, PhD^1)^.

**Affiliations:**

^1^Department of Emergency Medicine, Gunma University Graduate School of Medicine, Gunma, Japan.

^2^Department of General Medicine, Juntendo University, Tokyo, Japan.

^3^Department of Health Services Research, Faculty of Medicine, University of Tsukuba, Ibaraki, Japan.

^4^Department of Traumatology and Emergency Medicine, National Defense Medical College, Saitama, Japan.

Supplementary Table 1. Characteristics of traumatic pediatric patients with in-hospital death divided by age group

| Variable | Age  (<1)  n=34 | Age  (1-5)  n=142 | Age  (6-10)  n=141 | Age  (11-15)  n=179 |
| --- | --- | --- | --- | --- |
| Gender (male) | 24/34 (71%) | 91/142 (64%) | 97/141 (69%) | 119/179 (67%) |
| Cause of injury  Traffic accident  Fall  Fall from high place  Fall down  Fall at same level  Sport  Other blunt trauma  Penetrate  Burn | 9/29 (31%)  5/29 (17%)  2/29 (6.9%)  2/29 (6.9%)  0/29 (0%)  6/29 (21%)  3/29 (10%)  2/29 (6.9%) | 85/135 (63%)  23/135 (17%)  4/135 (3.0%)  3/135 (2.2%)  0/135 (0%)  13/135 (9.6%)  0/135 (2.7%)  7/135 (5.2%) | 106/140 (76%)  19/140 (14%)  3/140 (2.1%)  1/140 (0.7%)  5/140 (3.6%)  4/140 (2.9%)  1/140 (0.7%)  1/140 (0.7%) | 80/173 (46%)  58/173 (34%)  3/173 (1.7%)  2/173 (1.2%)  5/173 (2.9%)  16/173 (9.2%)  2/173 (1.2%)  7/173 (4.0%) |
| AIS (median [IQR])  Head (n=375)  Face (n=97)  Neck (n=9)  Thorax (n=252)  Abdomen and pelvis (n=86)  Cervical spine (n=66)  Upper extremity (n=89)  Lower extremity (n=152)  Others (n=39) | 5 (1)  1 (0)  N.A  4 (3)  3 (1)  N.A  2 (1)  3 (0)  5 (0) | 5 (1)  2 (1)  1 (0)  4 (1)  3 (2)  6 (3)  2 (1)  3 (2)  2 (4) | 5 (2)  1 (1)  3 (0)  4 (2)  3 (2)  2 (4)  2 (1)  3 (1)  1 (0) | 5 (2)  1 (1)  4 (6)  4 (2)  4 (3)  2 (2)  2 (0)  3 (2)  1 (4) |
| ISS (median [IQR]) | 25 (10) | 29 (29) | 29 (18) | 34 (25) |
| FAST  Positive  Negative  Not conducted  Pan-scan CT | 2 (6.3%)  22 (69%)  8 (25%)  6 (18%) | 11 (8.5%)  82 (64%)  36 (28%)  39 (28%) | 25 (19%)  89 (69%)  15 (12%)  50 (36%) | 32 (19%)  93 (55%)  43 (26%)  60 (34%) |
| Emergency procedure  Resuscitative thoracotomy  Resuscitative endovascular balloon occlusion of the aorta  Treatment  Craniotomy  Thoracotomy  Celiotomy  TAE  Blood transfusion | 0 (0%)  0 (0%)  7 (21%)  0 (0%)  0 (0%)  0 (0%)  17 (52%) | 10 (7.0%)  1 (0.7%)  11 (7.7%)  13 (9.2%)  6 (4.2%)  1 (0.7%)  42 (31%) | 6 (4.3%)  0 (0%)  24 (17%)  17 (12%)  12 (8.5%)  2 (1.4%)  72 (54%) | 15 (8.4%)  6 (3.4%)  11 (6.1%)  20 (11%)  13 (7.3%)  4 (2.2%)  63 (36%) |
| Mortality in ED | 9/34 (27%) | 58/142 (41%) | 42/141 (30%) | 72/179 (40%) |

IQR, interquartile range; sBP, systolic blood pressure; HR, heart rate; RR, respiratory rate; GCS. Glasgow coma scale; RTS, revised trauma score; AIS, abbreviated injury scale; ISS, injury severity score; TRISS, trauma and injury severity score; FAST, focused assessment with sonography for trauma; TAE, transcatheter arterial embolization; ED, emergency department.

Missing: Cause of Injury=19, ISS=39, FAST=38 and Blood transfusion=18.

Supplementary Table 2. Characteristics of traumatic pediatric patients with prehospital and/or arrival death divided by age group (years)

| Variable | Age  (<1)  n=17 | Age  (1-5)  n=89 | Age  (6-10)  n=70 | Age  (11-15)  n=109 |
| --- | --- | --- | --- | --- |
| Gender (male) | 11/17 (65%) | 55/89 (62%) | 48/70 (69%) | 70/109 (64%) |
| Cause of injury  Traffic Accident  Fall  Fall from high place  Fall down  Fall at same level  Sport  Other blunt trauma  Penetrate  Burn | 5/14 (36%)  0/14 (0%)  0/14 (0%)  0/14 (0%)  0/14 (0%)  5/14 (36%)  3/14 (21%)  1/14 (7.1%) | 60/84 (71%)  12/84 (14%)  0/84 (0%)  0/84 (0%)  0/84 (0%)  9/84 (11%)  0/84 (0%)  3/84 (3.6%) | 59/69 (86%)  6/69 (8.7%)  0/69 (0%)  0/69 (0%)  1/69 (1.4%)  3/69 (4.3%)  0/69 (0%)  0/69 (0%) | 40/105 (38%)  42/105 (40%)  1/105 (1.0%)  2/105 (1.9%)  0/105 (0%)  14/105 (13%)  1/105 (1.0%)  5/105 (4.8%) |
| AIS (median [IQR])  Head (n=201)  Face (n=58)  Neck (n=5)  Thorax (n=153)  Abdomen and Pelvis (n=46)  Cervical Spine (n=45)  Upper extremity (n=55)  Lower extremity (n=98)  Others (n=24) | 5 (4-6)  1 (1-1)  N.A  4 (4-5)  3 (2-3)  N.A  3 (2-3)  3 (3-3)  N.A | 5 (4-6)  2 (1-2)  1 (1-1)  4 (3-5)  3 (2-4)  6 (3-6)  2 (1-2)  3 (2-3)  1 (1-5) | 4 (3-5)  1 (1-2)  N.A  4 (4-5)  3 (2-4)  3 (2-6)  2 (1-3)  3 (2-3)  1 (1-1) | 4 (3-5)  1 (1-2)  N.A  4 (3-5)  4 (2-6)  3 (2-3)  2 (2-2)  3 (2-4)  1 (1-3) |
| ISS (median [IQR]) | 21 (9-45) | 40 (25-75) | 29 (25-50) | 34 (26-59) |
| FAST  Positive  Negative  Not conducted  Pan-scan CT | 2/15 (13%)  10/15 (67%)  3/15 (20%)  2 (12%) | 9/80 (11%)  50/80 (63%)  21/80 (26%)  24 (27%) | 15/62 (24%)  39/62 (63%)  8/62 (13%)  19 (27%) | 22 (22%)  52 (52%)  26 (26%)  30 (28%) |
| Treatment  Craniotomy  Thoracotomy  Celiotomy  TAE  Blood Transfusion | 0 (0%)  0 (0%)  0 (0%)  0 (0%)  5/16 (31%) | 0 (0%)  10 (11%)  5 (5.6%)  0 (0%)  17/87 (20%) | 0 (0%)  11 (16%)  6 (8.6%)  0 (0%)  20/66 (30%) | 0 (0%)  17 (16%)  6 (5.5%)  0 (0%)  26/106 (25%) |
| Mortality in ED | 8 (47%) | 51 (57%) | 39 (56%) | 66 (61%) |

IQR, interquartile range; AIS, abbreviated injury scale; ISS, injury severity score; FAST, focused assessment with sonography for trauma; TAE, transcatheter arterial embolization; ED, emergency department.

Missing: Cause of injury=13, ISS=29, FAST=28 and Blood transfusion=10.

Supplementary Table 3. Characteristics of all pediatric trauma patients between early and late periods

|  | Early period  (2004 – 2010)  n= 5,249 | Late period  (2011– 2015)  n = 10,192 | P value |
| --- | --- | --- | --- |
| Patients number according to age category (years)  Age < 1  Age 1– 5  Age 6– 10  Age 11– 15 | 274 (5.2%)  1,274 (24%)  1,981 (38%)  1,720 (33%) | 505 (5.0%)  2,659 (26%)  3,564 (35%)  3,464 (34%) | 0.003^*^ |
| Gender (male) | 3,700/5,248 (71%) | 7,004/10,188 (69%) | 0.025^*^ |
| Cause of injury  Traffic Accident  Fall  Sport  Other blunt trauma  Penetration  Burn | 2,440/5,042 (48%)  1,627/5,042 (32%)  274/5,042 (5.4%)  320/5,042 (6.1%)  94/5,042 (1.9%)  287/5,042 (5.7%) | 4,100/9,824 (42%)  3,473/9,824 (35%)  909/9,824 (9.3%)  736/9,824 (7.5%)  164/9,824 (1.7%)  442/9,824 (4.5%) | < 0.001^*^ |
| AIS (median [IQR])  Head (n = 6,836)  Face (n = 2,696)  Neck (n = 109)  Thorax (n = 2,082)  Abdomen and Pelvis (n = 1,866)  Cervical Spine (n = 630)  Upper extremity (n = 3,841)  Lower extremity (n = 3,493)  Others (n = 1,401) | 3 (2– 4)  1 (1– 2)  1 (1-2)  3 (3-4)  2 (2-3)  2 (2-3)  2 (1-3)  2 (1-3)  1 (1-3) | 3 (2– 4)  1 (1– 2)  2 (1-3)  3 (3-4)  2 (2-3)  2 (2-3)  2 (2-3)  2 (1-3)  1 (1-3) | < 0.001^†^  0.532^†^  0.442^†^  0.145^†^  0.287^†^  0.505^†^  < 0.001^†^  0.461^†^  0.037^†^ |
| Severe head injury (AIS ≧ 4)  Severe chest injury (AIS ≧ 4)  Severe abdominal injury (AIS ≧4) | 1,200 (23%)  260 (5.0%)  103 (2.0%) | 1,600 (16%)  404 (4.0%)  154 (1.5%) | < 0.001^*^  0.004^*^  0.038^*^ |
| ISS (median [IQR]) | 9 (5– 17) | 9 (4– 16) | < 0.001^†^ |
| FAST  Positive  Negative  Not conducted  Pan-scan CT | 326/4,662 (7.0%)  2,769/4,662 (59%)  1,567/4,662 (34%)  879 (17%) | 413/9,494 (4.4%)  4,819/9,494 (51%)  4,262/9,494 (45%)  2,360 (23%) | < 0.001^*^  < 0.001^*^ |
| Treatment  Craniotomy  Thoracotomy  Celiotomy  TAE  Blood Transfusion | 258 (4.9%)  37 (0.7%)  86 (1.6%)  91 (1.7%)  353/4,824(7.3%) | 294 (2.9%)  37 (0.4%)  105 (1.0%)  145 (1.4%)  496/9,748 (5.1%) | < 0.001^*^  0.004^*^  0.001^*^  0.136^*^  < 0.001^*^ |
| CPA on arrival at hospital  Mortality in ED  Mortality in hospital | 136 (2.6%)  95 (1.8%)  243 (5.5%) | 195 (1.9%)  121 (1.2%)  253 (3.0%) | 0.006^*^  0.002^*^  < 0.001^*^ |

IQR, interquartile range; AIS, abbreviated injury scale; ISS, injury severity score; FAST, focused assessment with sonography for trauma; TAE, transcatheter arterial embolization; ED, emergency department.

Missing: Gender = 5, Cause of injury = 575, ISS = 1,416, FAST = 1,285, Blood transfusion = 869, Mortality in ED = 44 and Mortality in hospital = 2,567

*Chi-Square test, †Man-Whitney U test.
